# Knowledge and practices of home caregivers on neonatal danger signs pre-admission to tamale teaching hospital, Ghana: an explorative descriptive study

**DOI:** 10.1186/s12887-023-03879-5

**Published:** 2023-03-30

**Authors:** Joyce Fatima Kanton, Alberta P. Gyepi-Garbrah, Olivia Nyarko Mensah, Doris Richardson, Dzigbordi Kpikpitse, Hannah Acquah, Stephanie Ajinkpang, Deborah Azalekor, Mary Ani Amponsah, Alhassan Abdul-Mumin

**Affiliations:** 1 Department of Paediatrics and Child Health, Tamale Teaching Hospital, P.O. Box TL 16 Tamale, Tamale, Ghana; 2 Ghana College of Nurses and Midwives, Accra, Ghana; 3 Child Health Department, 37 Military Hospital, Accra, Ghana; 4 Maternal and Child Health Department, School of Nursing and Midwifery, University of Ghana, Accra, Ghana; 5 Department of Pediatrics and Child Health, School of Medicine, University for Development Studies, Tamale, Ghana

**Keywords:** Caregivers, Danger signs, Health seeking behavior, Mother, Newborn illness

## Abstract

**Introduction:**

Neonatal illnesses can prove to be fatal if not identified early and treated. This suggests that death occasioned as result of neonatal illness could be prevented. However, it has been observed that most mothers report to the hospital late with their newborns in critical state, making it difficult for professionals to salvage the problem often than not. This study sought to explore the knowledge and practices of home caregivers on neonatal danger signs pre-admission to Tamale Teaching Hospital a tertiary hospital in northern Ghana.

**Methods:**

An explorative descriptive qualitative design was used in this study. Purposive sampling technique was used to select fifteen caregivers of neonates on admission at the Neonatal Intensive Care Unit of Tamale Teaching Hospital. Data was collected using semi-structured interview guide. As part of data collection, audio recordings were used to audio tape interviews. All data collected were transcribed verbatim and subsequently analyzed manually using thematic content analysis.

**Results:**

Thematic analysis in the study demonstrated that caregivers had basic knowledge, describing neonatal illness with danger signs such as lethargy, convulsion, fever, fast breathing, poor feeding, vomiting and diarrhea. The study further found that the predominant practice to care seeking by caregivers was home/traditional herbal remedies. It also indicated that inexperience caring for neonates, severity of illness and non-availability of finances were factors that informed caregivers choice of treatment of neonatal illness.

**Conclusion:**

The study concludes that inexperience caring for neonate, severity of illness and non-availability of finances were factors that informed caregivers choice of treatment. There is a pressing need for health workers to strengthen the education of caregivers/mothers on neonatal danger signs and the need for prompt care seeking from skilled health care providers prior to discharge from the hospital.

## Introduction

The neonatal period is the first 28 days in a person’s life, and this is a crucial time for child survival [1–3]. It represents the period when the newborn is susceptible to diseases and at risk of death [4]. Neonatal health is among the unachieved millennium development goal targets identified for the post-2015 priorities [5], with neonatal deaths still accounting for 44% of under five deaths globally.

According to World Health Organisation (WHO), 2.5million deaths are recorded within the neonatal period, which constitutes about 45% of all under-five deaths [6]. Sub-Saharan Africa alone accounts for more than 25% of global neonatal deaths and recorded the highest neonatal death rate of 28 deaths per 1000 live births in 2018 [6]. The high morbidity and mortality rates attest to the fragility of life during this period [7].

Hence, in response, several intervention programs have since been developed by international bodies to facilitate early identification of these health problems and reduce neonatal morbidity and mortality in several countries, including Ghana, some of which include: the Integrated Management of Newborn and Childhood Illness (IMNCI) developed by WHO which focuses on the assessment of general danger signs in the examination of children presenting with illness at health care centres [8]; Every Newborn Action Plan (ENAP) implemented by WHO and United Nations International Children’s Emergency Fund (UNICEF) Ministry of Health (MOH) [9]. In addition, WHO in 2013 strongly recommended specific danger signs that should be assessed during each postnatal care contact and that the newborn should be referred for further evaluation if any of the signs are present [10]. As if that is not enough, locally, Ghana implemented the National Newborn Health Strategy and Action Plan to reduce neonatal mortality [11]. This took place in June 2014 under the supervision of the MOH, GHS and development partners. The major goals of this national strategy and action plan were to drastically reduce the number of babies who die in the neonatal period from 32 per 1000 live births in 2011 to 21 per 1000 live births in 2018 and to reduce institutional neonatal mortality by at least 35% by 2018.

Despite the various initiatives that seek to end preventable newborn death by improving neonatal care services [11,12], millions of mothers and their newborns live in a social environment that does not support proper healthcare-seeking behaviour in sub-Saharan Africa [12]. Thus, many caregivers do not generally seek proper healthcare during childbirth and puerperium, which has significant impacts on the newborn’s health and survival [13]. Particularly, in Ghana, neonatal deaths have thus become an important component of under-five deaths, accounting for as high as 40% of under-five mortality, which constitutes a major bottleneck, preventing Ghana from achieving the set MDG 4 goal (9). In light of the above, this study sought to establish the knowledge and practices of home caregivers on neonatal danger signs pre-admission to the Tamale Teaching Hospital of Ghana.

## Methods

### Study design

The study used a descriptive qualitative research design to explore the knowledge and practice of home caregivers on danger signs of neonatal illness pre-admission at the Tamale Teaching Hospital. The qualitative approach was considered appropriate because it allowed the researcher to capture in-depth information based on the study participants’ experiences and knowledge about the danger signs of neonatal illness.

### Study setting

This study was conducted at the Neonatal Intensive Care Unit (NICU) of the paediatrics department of the Tamale Teaching Hospital (TTH) because as at the time of conducting this study, it was the only referral facility with an established NICU in the northern part of Ghana. TTH is situated in the Eastern part of Tamale, the capital of the northern region. It is the only teaching hospital in the northern part of Ghana and was established to serve as a primary medical referral Centre for the Northern, Upper East, Upper West Regions, the northern parts of the Brong Ahafo Region and the neighbouring countries of La Cote D’Ivoire, Burkina Faso and Togo.

### Target population and sampling

The target population for the study were caregivers’ (mothers) who had their neonates admitted to the NICU of Tamale Teaching Hospital. Participants who were 18 years and above and could express themselves in Dagbani or English and consented to taking part in the study were included in the study. However, caregivers who were psychologically traumatized, had post-delivery complications, were excluded in the study. Also, health workers who had their babies on admission were excluded. Participants who met the inclusion criteria were purposively sampled until data saturation was reached with the 15 participants.

### Data collection procedure

The NICU of the hospital was the outlet for recruitment; thus, permission was sought from the hospital authorities to conduct the study, and the hospital’s research department issued an authorisation letter to allow the researcher access to the study site for data collection. Nurses on the ward were informed about the purpose of the study clarifying the inclusion and exclusion criteria. It aided the nurses in identifying suitable participants that met the inclusion criteria for the study. Participants who met the inclusion criteria were primarily contacted by the researchers to establish rapport and then enrolled in the study after obtaining informed consent.

In-depth face to face interviews was conducted using a semi-structured interview guide designed by the researcher and reviewed by supervisors for content and flow of the questions. The design of the interview guide was done based on prior literature. The interview guide consisted of two sections. Section A constituted participants’ demographic data, while section B entailed open-ended questions and probing questions specific to the study’s objectives. In addition, as part of data collection audio recordings were used, with participants consent. All interviews were conducted in English or Dagbani because these are dialects both the researcher and participants understood and could speak fluently. Questions were asked concisely, and participants were encouraged to freely express their thoughts since there was no right or wrong answer. Interviews were conducted at a quiet place in the hospital and lasted between 20 to 35 minutes. Participants’ responses were extra probed or redirected during the interview to ensure that adequate and thorough responses were elicited from participants. Interviews were conducted by JFK. The researcher took field notes of all non-verbal communication to aid in data analysis to enhance understanding of data.

### Data analysis

Interviewed data from the study was analysed inductively and concurrently with data collection using thematic content analysis. This was done to ensure that all data followed a rational pattern. Interviews were manually transcribed verbatim immediately after each session. The precision of the transcript was ensured by reading through the transcripts while listening to the recorded audio. For data immersion, transcripts were read severally to comprehend the participants’ views fully. While reading through the transcripts, JFK and SA looked out for similar ideas and thoughts in the transcript and grouped them into meaning as codes. Subsequently, relations between codes were analysed, and similar codes were grouped into themes and sub-themes.

### Ethical consideration

Ethical clearance was obtained from the Ethics Review Committee of Ghana Health Service for ethical clearance, where the study was reviewed and approved (GHS-ERC 037/03/20); an authorisation letter was obtained from the Research Review Board of Tamale Teaching Hospital to pave the way for initiation of the data collection process. Written informed consent form which was approved by the Ethics Review Committee of Ghana Health Service was given to all caregivers who agreed to participate, and they were assured that their responses would be anonymous. No participant identifiers were collected.

### Rigour of the study

The trustworthiness of the study findings was improved through credibility, dependability, transferability, and confirmability. Credibility was ensured by structuring the interview guide to elicit accurate responses to questions using techniques like providing privacy and confirming responses if the interviewer was unsure of their meaning, credibility was established by conducting this research in a way that made confidence in the veracity of the information gathered during the interview evident. The required number of participants who could freely discuss their experiences was chosen via purposeful sampling. The researcher clarified specific statements made by the participants during the interviews by repeating them. Participants were asked to confirm their responses following the interview to ensure that the precise information supplied was disclosed in cases where the response was still uncertain. Each interview’s transcript was individually coded and categorised by the researchers.

Transferability is the degree to which data findings can be used or adapted to different groups or contexts is known as transferability. The ability of the results to be duplicated by other researchers was improved by a full description of the concept and methodology, the research setting at the Tamale Teaching Hospital, and the number of participants involved.

To attain confirmability, the researcher organized the study, kept field notes to document the activities involved in data collecting, and made sure that the data analysis was accurate and objective. For making sure that the results accurately reflected the participants’ perceptions and experiences, the researcher recorded and transcribed interviews verbatim.

To ensure dependability data analysis and codes were audited by an outside reviewer in order to improve dependability, or the stability of the data through time and under varying situations. In order to look for any discrepancies, this investigation audit also included pertinent supporting documentation and field notes. Furthermore, a complete explanation of the study context, methodology and data analysis in participants’ language was done to ensure that the reader appreciates the data from the participants and their worldview (thick descriptions).

## Results

A total of fifteen (15) female caregivers of sick neonates admitted at the NICU of Tamale Teaching Hospital were sampled for the study. The age distribution showed that most caregivers (14) were between 21 and 30 years of age, with one in the age bracket of 31-40 years. Out of the 15 sampled participants, a more significant percentage were Muslims (80%), with the minority being Christians (20%), all participants were married with children ranging from one to five. Most caregivers had secondary and tertiary levels of education.

**Fig. 1:**
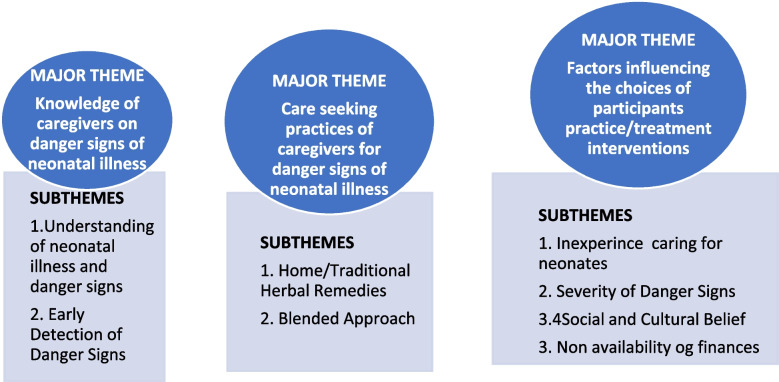
Major themes identified during the study

Three major themes and seven subthemes were identified in this study. The major themes are indicated in Fig. 1 below.

### Knowledge of caregivers on danger signs of neonatal illness

As indicated in Fig. 1, knowledge of danger signs of neonatal illness is an essential factor towards recognising serious illnesses in neonates; thus, maternal knowledge of danger signs during neonatal illness constitutes a significant factor in contributing to neonatal morbidity. Therefore, the study needed to establish caregivers’ knowledge regarding danger signs of neonatal illness. Two (2) indicators were considered in assessing the participants’ knowledge: Understanding Neonatal illness and Danger Signs and How early caregivers detect neonatal danger signs.

### Understanding of neonatal illness and danger signs

Generally, interview sessions with sampled participants established that caregivers had a basic knowledge of neonatal illness and danger signs. Most participants presented biomedical explanations of neonatal illnesses, describing neonatal illness using danger signs and symptoms such as predominantly refusing to feed, vomiting, convulsing, fever, lethargy, and diarrhoea. For instance, below is what a participant in the study had to say:

*“Neonatal illness is when the baby is not feeling well and starts vomiting or has a fever and convulsing”. (P15)* [30-year-old petty trader, First child mother].

Another participant added:

*“As for me, when my child is coughing and cannot breathe or is vomiting or running diarrhoea and having fever or jitteriness, especially at night, it is not normal and that my neonate is ill.*” *(P13)* [29-year-old civil servant, second child mother].

A third study participant expressed the following sentiments:

*“Neonatal illness is when a baby has high body temperature. Sometimes, the baby is over crying and refusing to feed, vomiting, and looking weak”. (P5)* [25-year-old unemployed first child mother].

### Early detection of danger signs

Regarding the timeliness in identifying illness in neonates, the findings indicate that caregivers noticed signs of illness in their neonates between one to 7 days following birth. The majority of sampled caregivers indicated that they noticed their neonate’s condition after being discharged from health centres after birth, which was within 24 hours, even though some study participants revealed they were not privileged to give birth in the hospital. The following narrative captures the view of a caregiver on how neonatal illness presented in her neonate:

*“A day after discharge, I noticed my baby’s skin colour had become very pink, which was unusual. However, on the third day, I was alarmed when I noticed that my baby’s eyes were yellow. This made me seek immediate care for my baby”. (P6).* [32-year-old head dresser, second child mother].

#### Other participants added

*“Three days after discharge from the hospital, I noticed my baby was warm to touch and began to have fast breathing, making it difficult to feed when I attach baby to breast”. (P15)* [28-year-old seamstress, first child mother].

*“I gave birth at the hospital and was discharged home few hours after delivery, a day after discharge, I notice my baby was inactive, had jerky movement, and refused to feed when I put to breast”. (P7)* [26-year-old unemployed first child mother].

However, for those caregivers who gave birth at home, the timeliness of detection of neonatal illness was delayed. A significant number of them took some of the signs as normal amongst newborns and therefore would only take such signs seriously when their expectations are becoming unusual. Below is an account of one of the caregivers on the timely detection of neonatal danger signs:

#### A 39-year-old farmer in her fifth time of having a child had this to say

*“My current baby is the fifth born and was delivered at home. Three days after birth, my baby became irritable, which is common with my children. But on the fifth day, I noticed the base of my baby’s cord was red, and when I touched the knees, my baby would cry more. Baby’s knees were warm to touch, so I informed my husband, and we sought care for my baby”. (P14).*

#### Another participant added

*“I gave birth at home, and five days later, I noticed that the eyes and skin colour of my baby had changed, but I did not know that my baby was ill until he started having fever and high pitch cry after the naming ceremony, so my husband and I decided to visit the hospital” (P8).* [31-year-old petty trader, first child mother].

### Care seeking practices of caregivers for danger signs of neonatal illness

This part of the study sought to establish the care-seeking practices of caregivers for danger signs of neonatal illnesses (Fig. 1). Thus, eliciting participants’ views on the choice of interventions. Generally, the study revealed that participants were engaged in different practices in their attempt to treat the illness of their neonates. These practices were broadly grouped into two, namely: Home/Traditional Herbal Remedies and the Blended Approach (Home/Traditional Herbal Remedies and Pharmaceutical Medicines).

### Home/traditional herbal remedies

Essentially, the findings regarding this inquiry revealed that caregivers predominantly adopted home/traditional treatment using herbs. According to the participants, these remedies were typical and age-old for them. They were comfortable and had no qualms at all adopting it. However, some of the participants revealed they had no option but to follow the ways of the family for which they were married, though they were not happy with the treatment choice of the neonates.

#### A 25-year-old unemployed participant with her first child had this to say

*“When my baby had difficulty passing stool on the fourth day after birth, his grandmother started to give him grounded herbs “musuro and kanafir“ as an enema. With this, my baby started passing stool very well. She also used warm water in an empty tin container with a hole under it and then applied it on the anus after the enema had been given”. (P2).*

#### Another participant, 39 years old farmer with fifth son added

*“Buying drugs from the drug store is a common practice that many people engage in at first hand even before they think of going to the hospital. As for the use of herbs, I learnt this from my aunty growing up and it always worked for her, so I also use them whenever my child is ill”. (P4).*

#### Other participants had this to say

*“When I noticed my babies were feeding poorly and looking lethargic, I informed my husband, and he suggested we gave some burnt herbs to the babies to help boost their appetite and clean any dirt in their stomach”. (P12)* [*39 years* petty trader with her fourth child].

*“I decided to try sunbathing my baby at home for three days when I noticed his skin was becoming yellow; I was also advised to apply breastmilk into my baby’s eyes to clear the yellowish colour from the eyes.” (P10)* [28 years old, apprentice make-up artiste with second child].

### Blended approach

The findings further revealed that some caregivers utilised a blend of traditional and pharmaceutical medicines to intervene following the detection of danger signs of neonatal illnesses. Hospital intervention for neonatal illnesses was sought, particularly in situations where home remedies such as herbal treatment and pharmaceutical medicine did not effectively treat neonatal illnesses.

#### According to some participants

*“My baby had fever on the fourth day after delivery. I gave her suppository paracetamol, but the fever resurfaced the next day, so I bathed and massaged my baby with herbs to reduce the fever and prevent convulsion. I also used garlic to smear my baby’s body to reduce the fever and applied “allaigi“ (palm kennel oil) in my baby’s nostrils to prevent him from catching a cold, but when I did not see any improvement in the baby’s illness, I reported to the hospital.” (P1)* [33-year-old petty trader, third child mother].

*“Since I got married into this family, I have realized that anytime a child is sick in the house what we do is to buy drugs from a chemical shop or those men who roam with drugs from house to house or use local herbs, we only go to the hospital when the condition does not get better”. (P9)* [27-year-old seamstress, second child mother].

The above narratives revealed that pathways for seeking care by caregivers for neonatal illnesses varied from one participant to the other. These variations ranged from home/traditional herbal medicine to professional services either from pharmacy shops or a health facility, and a blend of these two approaches.

### Factors that influenced participants care seeking decisions

As part of efforts to explore caregivers’ knowledge and health-seeking behaviour on danger signs of neonatal illness at Tamale Teaching Hospital, this section of the study sought to understand the factors that influenced participants’ care-seeking decisions. The study’s findings established that inexperience and the severity of illness were the major factors that influenced the participants care seeking decision with very few participants reporting that the non-availability of finance also influenced their care seeking decisions.

### Inexperience caring for neonates

Participants in accounting for the reason inexperience was a factor that influenced their choice of treatment decision, indicated that because most of them (participants) were first-time mothers, it was only prudent that they took advice from mothers-in-law, parents, other family members and friends who had some experience of a sought in childbirth and care. According to participants, this played a significant role in arriving at the care choice for caregivers during neonatal illnesses. The narratives below reveal the expressions of some of the participants on the above themes:

Below is what Participant 8, a 27-year-old unemployed first-time mother had this to say:

*“Because I am a first-time mother, I took suggestions from relations, especially my senior husband’s wife, who witnessed similar signs in her then neonate. She told me that such signs, as my neonate experienced, are typical with neonates and that I should remain calm and never visit the hospital but rather try some home remedies. She told me to give the baby some gripe water, and all will be fine. Lo and behold, after giving the gripe water, my baby was able to feed well and be active. So, I can say she influenced my choice of treatment practice” (P8).*

In the words of another participant [28-year-old teacher in her first time of giving birth], she stated:

*“Sometimes suggestions from experienced mothers influence treatment choice, especially when it comes to neonatal illness. Particularly in my case, my neonate frequently vomited, but I felt she was not hungry, but my mother-in-law, who is very educated, quickly sanctioned me to visit the hospital immediately. So, I can imagine her influence!” (P11).*

A third participant [25-year-old unemployed first-time mother] expressed the following sentiment regarding the influence of friends with regards to the choice of initial treatment for neonatal illnesses:

*“As for me, because this is my first time in childbirth, I easily get worried about any abnormality I get to witness in my neonate. So, anytime I see any changes in my child’s condition, I call a friend nurse to advise me on what to do. Therefore, I can say she usually influences the treatment choice” (P3).*

### Severity of danger signs

As an influencing factor, participants generally indicated that the severity of neonatal illnesses further informed their initial treatment choice. From the study, most caregivers believed that some neonatal illnesses such as fever, diarrhoea, vomiting, and malaria could be managed easily. However, if the situation is not improved and becomes severe, participants further revealed they become alarmed and begin looking for ways to find treatment for the worsening illnesses.

A participant [31-year-old teacher, first time mother] had this to say concerning severity as an influence:

*We took my baby to the hospital because I thought the baby was not getting any better using the home treatment, so the seriousness of the sickness prompted us to go to the hospital. So, I can say the severity of the illness influenced me to choose the hospital as my treatment choice (P3).*

### Social and cultural beliefs

Social and cultural beliefs of participants was also another important factor that influenced their treatment decision. According to some of the participants they lived in societies that have ways of doing things either socially or culturally. Therefore, it was obvious that some of these beliefs influenced their care seeking decisions. Socially, a few of the participants had the belief that it was normal for a neonate to show some ill signs like vomiting, and so there was no need to report such situations to the hospital for treatment. Below is what one of the participants [29-year-old unemployed, third time mother] had to say:

*See madam, if your child just shows some common ill signs like vomiting or fever and take him or her to the hospital, the hospital people will admit you and spend all your money mean while you can just treat them easily at home. (P12).*

However, regarding participants’ cultural beliefs in influencing their care seeking decision a 24-year-old unemployed first-time mother had this to say:

*“Because of cultural belief like in my case, my father-in-law had to insist we consult to see whether the illness is for the hospital or can be treated at home or be left to go on its own, and I think that is influential in my treatment choice”. (P4).*

### Non-availability of finance

For the few participants who revealed that finance was an influential factor in determining treatment choices, they indicated that it is hard to come by money these days, and so one cannot send a neonate to places like the hospital, the pharmacy shops etc. Who always demand money before treatment is done. Below is what a participant said in the narration below:

*“The hospital is good, but it is costly to seek treatment there if you are not financially sound. See, as for me, I do not have any meaningful work doing and my husband does not like visiting the hospital, so he is not supportive in that regard. As a result, any time my neonate is ill, I just use the local home treatment methods available” (P4).* [32-year-old unemployed fourth time mother].

#### Other participants [29-year-old unemployed first-time mother] added that

*“My poor financial situation resulted in me resorting to traditional herbal treatment after I detected that my baby had a temperature and experienced difficulty passing stool since my husband did not support the idea of seeking hospital treatment.” (P14).*

*“On my own, I do not have any source of income. So, my husband takes care of all the family’s needs, including children’s healthcare. If he does not have money or is unwilling to cover hospital care, I am forced to try home remedies”. (P6)* [31-year-old unemployed second child mother].

## Discussion

### Knowledge on danger signs of neonatal illness

Neonatal danger signs are so critical that newborns who suffer danger signs after delivery are two times more likely to die than those who never suffered any danger sign [14]. Therefore, this study sought to explore the knowledge on danger signs of neonatal illness. Generally, this study found out that caregivers had basic knowledge of the danger signs of neonatal illness since they could describe neonatal illness using danger signs and symptoms. Some of the predominantly mentioned signs and symptoms were poor feeding, vomiting, convulsing, fever, lethargy, difficulty breathing and diarrhoea. Thus, a significant number of the study participants were able to mention at least one of the neonatal danger signs as recommended by WHO, which is similar to [15–17] who found in their respective studies that the majority of their study participants were able to mention one of the WHO recommended danger signs of neonatal illness. Contrarily, studies in Nigeria, Kenya, and Ethiopia respectively showed that mothers’ knowledge of neonatal danger signs was low [18–20]. This, therefore, implies that stakeholders in the health sector, particularly in Africa, still have some more work to do, especially in ensuring that the knowledge of caregivers on neonatal danger signs is improved and subsequently, reduce neonatal deaths in order to meet the Sustainable Development Goal (SDG) sub-goal 3.2 of goal three (3) which seeks to end preventable neonatal deaths and deaths in children under 5 years by 2030 [21, 22]. Regardless, the tendency to miss this goal is imminent as the results of this study further revealed that newborn mothers are discharged earlier from the hospital before the recommended 24-hour postnatal care for newborn mothers [23]. This makes it difficult for health workers, particularly nurses, to detect neonatal illnesses after discharge of caregivers since they (nurses) are unable to fully examine the newborn as recommended by WHO (2014) best practices.

### Care seeking practice of caregivers on neonatal danger signs

Evidence suggests that a significant proportion of neonatal mortality can be prevented by inexpensive, simple practices and interventions along the continuum of care starting from pre-pregnancy, during pregnancy, delivery, and postnatal period [24]. In assessing the care-seeking practices of caregivers, it was established in this study that caregivers predominantly utilised home/traditional herbal treatment, pharmacy shops, and hospitals for diagnosis and treatment of danger signs of neonatal illness. However, home treatment using traditional herbal medicine was the most preferred care seeking practice adopted by caregivers, followed by medicine obtained from drug stores or pharmacies, similar to the studies by [25, 11, 26] who found in their independent studies that caregivers upon detecting neonatal danger signs amongst their wards or children resorted to the use of various home and traditional herbal treatment. According to these scholars, home treatment was not done anyhow. They indicated that the use or choice of treatment depended upon how these danger signs were manifested in the neonate, which was corroborated in this study. For instance, according to [25], sick neonates with convulsions were given neem juice, and hypothermic babies were given mustard oil massage.

On the contrary, however, [27, 28] revealed in different studies respectively that participants in their study sought health care for their neonates from health posts and health centres immediately upon witnessing any neonatal danger signs. This practice has the potential to improve not only the health of neonates but increase their chances of survival compared to the practice of seeking health care for neonates using home and other traditional self-treatments methods since traditional care practices with the use of herbs and other self-medication during delivery and immediate care for a newborn at home and in the community inevitably affect the health of the neonates [24]. Therefore, there is the need for the practice of having to seek medical care at the hospital or any other health facility where professional examination, diagnosis, and treatment could be accessed.

### Factors that influenced participants care seeking decisions

Considering that most participants were first-time mothers, it was not unexpected that their choice of initial treatment was informed by relations and friends, which the pathway way model describes as “significant others” of which its importance in the model is much stressed [29, 30]. Though contrary [31] who acknowledged that the most common influence on the choice of neonatal care is the availability of finances, this study is in tandem with [32, 11] who also found that caregivers often received support from their family members in decision-making regarding care-seeking for neonatal danger signs. Arguably, the support from family members in the choice of care-seeking could yield either positive or negative treatment outcomes. However, in situations where home remedies including divinity are chosen by caregivers with support from family members, which is often the case [33], treatment outcomes may be difficult to predict in the absence of verified diagnosis of danger signs of neonatal illness, and this is what causes severity in neonatal illness like some participants revealed in the study. This according to participants, caused their delays in going to the hospital, congruent with the assertion by, [34] that caregivers particularly in their decision to manage neonatal illness is influenced by their perception of its severity.

### Strengths and limitations

Qualitative studies approach provides the opportunity to explore the knowledge and practices of home caregivers on neonatal danger signs pre-admission to the hospital further in-depth information than would be applicable in quantitative studies. Although this may affect generalizability to all caregivers, qualitative studies are likely to offer direction on what to explore or consider in a subsequent quantitative approach. The study used adult participants in exploring the knowledge and practices of home caregivers on neonatal danger signs pre-admission to Tamale Teaching Hospital. In view of this, replicability of the study’s findings may be limited to groups that shared similar features as the study sample. Furthermore, the interviews were conducted at the time participants were on admission at participant’s own convenience. However, there were times when interviews were interrupted by relatives of participants and healthcare providers. Such interruptions affected the chain of narration by participants during interviews. In this study, recall bias was possible because the data are based on the retrospective experiences of the participants, and some may have difficulties in recalling vividly exactly the issue they experienced.

## Conclusion

The study suggested that caregivers’ knowledge of danger signs of neonatal illness was appreciable. Generally, the study also revealed that participants predominantly engaged in different practices such as home/traditional remedies and visiting the hospital in their attempt to treat the illness of their neonates. The study revealed inexperience caring for neonates and severity of illness as the major factors that informed most caregivers’ choice of treatment, with a few of them mentioning the non-availability of finances. Therefore, it is imperative to reinforce the education of expectant mothers on danger signs of neonatal illness and the need to seek appropriate health care services from skilled professionals, promote institutional delivery, and improve neonatal care services during antenatal care and postnatal care follow-up visits.

## Data Availability

The data sets generated during the current study are available from the corresponding author upon request.

## Abbreviations

WHO: World Health Organisation
ENAP: Every Newborn Action Plan
IMNCI: Integrated Management for Neonatal and Childhood Illness
UNICEF: United Nation International Children Emergency Fund
TTH: Tamale Teaching Hospital
NICU: Neonatal Intensive Care Unit
MOH: Ministry of Health
SDG: Sustainable Development Goal
GHS: Ghana Health Service
